# Adolescent smoking, alcohol use, inebriation, and use of narcotics during the Covid-19 pandemic

**DOI:** 10.1186/s40359-022-00756-1

**Published:** 2022-02-26

**Authors:** Sabina Kapetanovic, Birgitta Ander, Sevtap Gurdal, Emma Sorbring

**Affiliations:** 1 Department of Social and Behavioral Studies, University West, Gustava Melinsgata 2, 46132 Trollhättan, Sweden; 2 Department of Psychology, Stockholm University, Stockholm, Sweden; 3 School of Health and Welfare, Jönköping University, Jönköping, Sweden

**Keywords:** Adolescents, Covid-19, Substance use, Parenting, Peer involvement

## Abstract

**Background:**

The aim of the study was to investigate how general family relations, reported changes in family interaction and involvement with peers during the Covid-19 pandemic, and following rules and recommendations during the pandemic relate to adolescent smoking, alcohol use, inebriation, and use of narcotics during Covid-19.

**Methods:**

An online national survey of Swedish adolescents (n = 1818) aged 15–19 years was conducted in June 2020. Hierarchical regression analysis was used to predict adolescents’ reported change in substance use during the pandemic. Person-oriented analyses, were used to identify clusters of participants characterized by similar patterns of substance use following ANOVA analysis with Scheffe post hoc tests testing differences between clusters in terms of family relations, reported changes in family interaction and involvement with peers during the Covid-19 pandemic, and following rules and recommendations during the pandemic.

**Results:**

Higher general family conflict, increased involvement with peers, a strained relationship with parents, and less compliance with rules and restrictions during the pandemic predicted a reported increase in adolescent substance use during this period. The grouping of scores for adolescent smoking, alcohol use, inebriation, and use of narcotics resulted in a six-cluster solution. One cluster (n = 767) either did not use or had decreased use of substances during the Covid-19 pandemic. Five other clusters, thus risk clusters, had retained or increased use of substances during the pandemic. Poor general family relations, increased peer involvement, and difficulties to conform to the rules and restrictions during the covid-19 pandemic were characteristics of risk clusters.

**Conclusions:**

Most of adolescents in our study did not increase their substance use during the pandemic. However, adolescents with poor family relations who turn to peers during stressful times and who have difficulty following the government’s rules and restrictions, are at risk of increased substance use during the pandemic. This is a potential threat both to adolescents themselves and others in their surroundings which is why at-risk adolescents and their families need more attention from public health and social services during this time of crisis.

As of December 2019, the world has been hit hard by the Covid-19 virus causing a state of global pandemic. The Swedish strategy to contain the virus was less invasive than in many other countries with no general lockdown, although as of March 17th, 2020 upper secondary school as well as universities moved to distance education, structured activities got cancelled, and social distancing alerts became common [1]. Some adolescents have had difficulties in their psychosocial adjustment, including involvement with substance use. Although reports from The Swedish Council for Information on Alcohol and Other Drugs indicate that the alcohol consumption among adults in Sweden in general does not seem to have increased [2], scholars, as well as public health professionals, have expressed concerns about a potential increase in substance use among adolescents during the pandemic [3], possibly as a means of coping with the difficult situation [4] or as a strategy for dealing with boredom [5]. This is not surprising given that adolescents do not have the same access to organized out of school activities, at the same time as schools are closed and access to school counsellors or other mental health services is limited. In essence, adolescent resilience when it comes to challenges brought about by the Covid-19 pandemic may be at risk [6], which may have adverse impact on their psychological health, including substance use.

Adolescent use of tobacco, alcohol and narcotics is a global health problem [7]. For example, The European School Survey Project on Alcohol and other Drugs (ESPAD) shows that among European 15–16-year-olds, at least 10% report daily cigarette use [8]. While the prevalence of cannabis use in the last 12 months is 13%, the use of alcohol is much higher. On average 79% report having consumed alcohol at least once during their lifetime and 13% report alcohol inebriation during the 30 days prior to the survey. In Sweden alone, 42% of 15-year-olds and 69% of 17-year-olds report having consumed alcohol during the last twelve months [9]. Although some experimentation with substances could be seen as a natural part of growing up [10], adolescent substance use may lead to more serious mental health problems including depression or substance abuse disorders [11]. In that sense, more attention to adolescent substance use and the factors that may explain such use are needed.

What characterizes adolescents with exacerbated use of alcohol, as well as other substances, such as tobacco and narcotics during the pandemic is yet to be understood. Developmental theories [12] suggest that parent–child relationships [13], including close bonds between parents and children [14] and parental knowledge of adolescents’ whereabouts [15], as well as adolescent involvement with peers [16] are critical social factors that have an impact on adolescent behavior and adjustment, including substance use. Indeed, one Swedish study of 755 adolescents and the trajectories of their alcohol and drug use, as well as criminality, suggested that poor family cohesion and engagement with deviant peers, are important explanatory factors for problematic behavioral development, including substance use issues [17]. Even during the Covid-19 pandemic, involvement with peers has been suggested as an important risk factor for adolescent use of substances [3]. Moreover, trust in the government’s way of handling the pandemic in general seems to have had an impact on individuals’ adjustment to health and behavior rules and recommendations during the pandemic [18]. Therefore, we will investigate to what extent general family cohesion, family conflict, parent-adolescent connectedness, reported changes in family interactions, and involvement with peers, as well as adolescents’ compliance with rules and recommendations during the Covid-19 pandemic are related to adolescents’ substance use during this time. In addition, using person-oriented analyses [19], we will investigate the characteristics of adolescents’ substance use profiles in terms of general family relations, reported changes in social relations during the Covid-19 pandemic, as well as adolescents’ compliance with rules and recommendations during the Covid-19 pandemic.

## Methods

### Sample and procedure

The project COVIDung (i.e. COVIDyoung) was designed to recruit adolescents aged 15 to 19 to study adolescents’ social relations, individual characteristics, and psychosocial changes during the COVID-19 outbreak in Sweden [20]. Between 8th June and 7th July 2020, we conducted a national online survey of adolescents aged 15–19 years using EasyQuest. Advertisements for the study were posted mainly on Instagram (70%), as well as Facebook and Twitter. The online survey contained a letter of information about the project, adolescent rights as research participants, as well as a request for informed consent. The survey took approximately 15–20 min to complete.

A total of 1818 adolescents completed the survey. Data from 51 adolescents were removed because they reported being younger than 15 or older than 19 years of age, and additional data from 123 adolescents were excluded because they failed to answer questions relating to substance use during the Covid-19 pandemic. Thus, the analytical sample consisted of 1644 adolescents (51.4% females and 48.6% males), born 2001–2005 (Md = 2003). In our sample, 71.5% were students in upper secondary school (ages 17–19) who mainly had distance education, and 28.5% were students in lower secondary school (ages 15–16) who had regular education in school.

### Measures

The measure Experiences related to Covid-19 [21] was used to assess changes in adolescent substance use, family relations and peer involvement during the Covid-19 pandemic. Adolescents were asked to rate the perceived changes in their own tobacco use, alcohol use, inebriation and use of narcotics, as well as changes in quality time spent with family, family conflict, hanging out on the streets without parents’ knowledge, and meeting with friends during the Covid-19 pandemic measured on a 5-point Likert scale ranging from 1 (decreased a lot) to 5 (increased a lot). The participants could also respond 0 for “I did not do this before the outbreak and have not started.”

General family climate [22] was used to measure family cohesion and conflict measured on a 4-point Likert scale, ranging from 1 (not true at all) to 4 (very true). Family cohesion was assessed with six items, such as “Family members really help and support one another” α = 0.83. Family conflict was assessed with five items, such as “We fight a lot in our family” α = 0.64.

General parent-adolescent connectedness [23] was used to assess adolescent perceived emotional connectedness to their mother and father respectively, measured with items such as “I feel comfortable sharing my private thoughts and feelings with my mother/father” measured on a 3-point Likert scale from 1 (Yes) to 3 (No, never). Reports for mother α = 0.89 and father α = 0.93 were subsequently averaged.

Adolescents also scored the item “I complied with the rules and suggestions of the government and health care system to remain at home to try to contain the virus” on a 4-point Likert scale ranging from 1 (do not agree at all) to 4 (agree completely) as a measure of adolescent compliance with rules.

### Statistical analysis

We performed the data analyses in four steps. First, we performed bivariate Pearson correlation analyses using SPSS, and second, we conducted multivariate hierarchical regression analyses in four stages with adolescent substance use as the dependent variable. Gender and age were entered at stage one as control variables. General family cohesion and control, as well as general parent-adolescent connectedness were entered at stage two, reported changes in social relations during the Covid-19 pandemic at stage three, and adolescents’ compliance with rules and recommendations during this period at stage four.

Next, we applied cluster analytic techniques to identify clusters of participants characterized by similar patterns of substance use using ROPstat, the statistical package for pattern-oriented analysis [24]. Before cluster analysis, we transformed all index scales into z-scores and analyzed the data to identify multivariate outliers. As no outliers were found, we could use the whole data set in the analyses. We applied Ward’s hierarchical clustering method to determine the number of clusters. To optimize the homogeneity of the chosen cluster the K-means clustering method was subsequently applied. We evaluated the cluster solution based on the following [25]: (1) level of homogeneity in the cluster solution (HC =  < 1), (2) the level of distance of cluster cases (HCmean =  < 1), (3) the extent to which cases are closer to their own cluster centers than to the total sample center EESS % which preferably should be above 67% [24]. We also accounted for the theoretical meaningfulness of the clusters. To compare clusters on variables of interest, in the final step of data analyses, we used one-way analysis of variance (ANOVA) and the Scheffe post hoc test.

## Results

Table 1 shows Pearson’s bivariate correlations between adolescent substance use (smoking, alcohol use, inebriation, and use of narcotics), general family relations (family cohesion and conflict), changes in social relations during the Covid-19 pandemic (quality time spent with family, conflicts with family, meeting with friends, hanging out without parents’ knowledge) and adolescent compliance with rules and restrictions during pandemic. Adolescent substance use was related to lower levels of general family cohesion and closeness and to following the rules and restrictions during the Covid-19 pandemic, as well as to higher levels of general family conflict. Similarly, adolescent substance use was related to a perceived decrease in quality time spent with family and perceived increase of family conflict and meeting friends during the pandemic.

**Table 1:** Correlations among study variables

	1	2	3	4	5	6	7	8
1. Adolescent substance use	–							
2. Gen. family cohesion	− .15**	–						
3. Gen. family conflict	.17**	− .62**	–					
4. Gen. closeness	− .09**	.62**	− .40**	–				
5. Fun times with family during C19	− .09**	.27**	− .18**	.25**	–			
6. Conflicts with family during C19	.10**	− .31**	.33**	− .20**	− .06*	–		
7. Meeting with friends during C19	.20**	.04	.01	.03	.09**	− .03	–	
8. Hanging out^a^ during C19	.32**	− .16**	.14**	− .14**	− .06*	.17***	.28**	–
9. Following restrictions^b^ during C19	− .25**	.09**	− .11**	.04	.07**	− .04	− .21**	− .22**

* < . 05 ** < .001; Gen = general; C19 = Covid-19

^a^Hanging out without parents’ knowledge

^b^Following rules and restrictions

As shown in Table 2, the results following hierarchical regression analyses revealed that at stage one, adolescent age contributed significantly to the regression model (β = 0.24 *p* < 0.001) and accounted for 6% of the variation in adolescents’ reported changes in substance use (R^2^ = 0.06 *p* < 0.001). Introducing general family relations at stage two, with general family conflict as a significant predictor (β = 0.14 *p* < 0.001) explained an additional 3% of variation in adolescents’ reported changes in substance use? (ΔR = 0.03 *p* < 0.001; R^2^ = 0.09 *p* < 0.001). Adding adolescents’ reported change in social relations during the pandemic to the model at stage three, with adolescents’ reported change in quality time spent with family (β =  − 0.09 *p* < 0.001), meeting with friends (β = 0.15 *p* < 0.001), and hanging out without parents’ knowledge (β = 0.28 *p* < 0.001) accounted for an additional 13% change of variation in adolescents’ reported changes in their use of substances during the pandemic (ΔR = 0.13 *p* < 0.001; R^2^ = 0.22 *p* < 0.001). Finally, adding adolescents’ compliance with rules and restrictions during the pandemic (β =  − 0.13 *p* < 0.001) explained an additional 2% of variation in adolescents’ reported changes in their use of substances during this time (ΔR = 0.02 *p* < 0.001; R^2^ = 0.24 *p* < 0.001). Together, all independent variables explain 24% of variation in adolescents’ reported changes in substance use during this period. Controlling for age and gender, higher general family conflict, increased involvement with peers and hanging out without parents’ knowledge as well as decreases in quality time with family and lower levels of compliance with rules and restrictions during the pandemic predicted a reported increase in adolescents’ substance use during this time.

**Table 2:** Hierarchical regression analysis of predictors of reported changes in adolescent substance use during Covid-19

	Model 1			Model 2			Model 3			Model 4		
	B	SE	β	B	SE	β	B	SE	β	B	SE	β
Gender	− .07	.06	− .03	− .07	.07	− .03	.04	.05	.02	.05	.05	.02
Age	.27	.03	.24**	.26	.03	.24**	.28	.02	.27**	.28	.02	.26**
*General family relations*												
Family cohesion				− .12	.07	− .06	− .04	.07	− .02	− .03	.07	− .01
Family conflict				.32	.07	.14**	.25	.07	.11**	.23	.07	.10**
Closeness				.00	.04	.01	.03	.03	.03	.03	.03	.02
*Change in social relations during Covid-19*												
Fun time with family							− .08	.02	− .09**	− .08	.02	− .08**
Conflicts in family							.02	.02	.03	.02	.02	.03
Meeting with friends							.13	.02	.15**	.11	.02	.12**
Hanging out without parents’ knowledge							.20	.02	.28**	.18	.02	.26**
**Restrictions during Covid-19**												
Following rules and restrictions										− .22	.04	− .13**
R^2^	.06**			.09**			.22**			.24**		

To investigate characteristics of adolescents who reported increases or decreases in their substance use during the pandemic we conducted hierarchical cluster analyses. The grouping of scores for adolescent smoking, alcohol use, inebriation, and use of narcotics resulted in a six-cluster solution. We based the chosen solution on homogeneity in the clusters (HC = 0.73–1.80), level of distance of cluster cases (HCmean = 0.31) and explained variance (EESS % = 84.49%). Although one of the clusters was above the preferred limits (HC = 1.80), we determined that the theoretical meaningfulness of the cluster was justified. Therefore, we settled on a solution where one cluster had somewhat violated homogeneity.

**Fig. 1:**
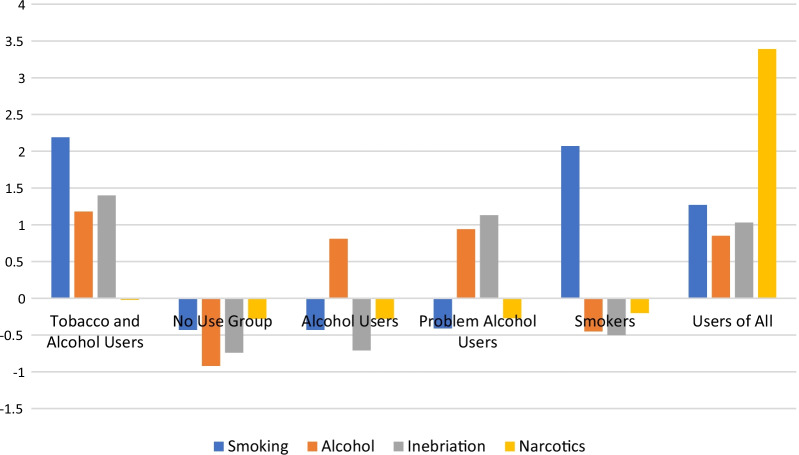
Six clusters describing adolescent substance use during the Covid-19 pandemic

The clusters had the following characteristics. As shown in Fig. 1, Cluster 1 (n = 114; 48.2% boys) consisted of adolescents who reported that their smoking, alcohol use and inebriation was either unchanged or had increased during the Covid-19 pandemic. We called this cluster Tobacco and Alcohol Users. Cluster 2 (n = 767; 50.7% boys) was the largest cluster consisting of adolescents who reported no use or decreased use of all substances during the Covid-19 pandemic. We called this cluster the No Use group. Cluster 3 (n = 169; n = 41.4% boys) consisted of adolescents who had unchanged or increased alcohol use, yet no use or decreases in smoking, inebriation, and use of narcotics during the pandemic. We called this cluster Alcohol Users. Cluster 4 (n = 390; n = 44.1% boys) consisted of adolescents with unchanged or increased alcohol use and inebriation during the Covid-19 pandemic, and no use or decreases in smoking and use of narcotics. We called this cluster Problem Alcohol Users. Cluster 5 (n = 76; n = 40.8% boys) consisted of adolescents who had unchanged or increased smoking, yet no use or decreases in alcohol use, inebriation and use of narcotics. We called this cluster Smokers. Cluster 6 (n = 120; n = 64.2% boys) mainly consisted of adolescents with unchanged or increased levels of all substance use, including smoking, alcohol use, inebriation and use of narcotics. We called this cluster Users of All. Most students from lower secondary school (60.5% out of n = 397) were in Cluster 2. Other clusters consisted mainly of adolescents from upper secondary school.

Table 3 shows mean levels and significant differences between clusters. The following significant differences in general family relations emerged among different clusters. Adolescents in Cluster 1 and 6, that is Tobacco and Alcohol Users cluster and Users of All cluster, reported lower levels of family cohesion in comparison to adolescents in Cluster 2 and 4, thus the No Use and Problem Alcohol Users clusters. Moreover, adolescents in the Users of All (6) cluster reported higher levels of family conflict than adolescents in the No Use (2), Alcohol Users (3) and Problem Alcohol User (4) clusters. In addition, adolescents in the Tobacco and Alcohol Users cluster (1) had significantly higher levels of family conflict than adolescents in the Problem Alcohol Users (4) cluster. Finally, adolescents in the Users of All (6) cluster reported lower levels of parent–child closeness in comparison to adolescents in the No Use (2) and Problem Alcohol Users (4) clusters. A significant difference also appeared among adolescents in the Tobacco and Alcohol Users (1) and Problem Alcohol Users (4) clusters where Tobacco and Alcohol Users (1) reported lower levels of parent–child closeness than adolescents in the Problem Alcohol Users (4) cluster.

**Table 3:** ANOVA results including means and standard deviations for independent variables among adolescents in different substance use clusters

Independent variables	Sample mean and sd^a^	Tobacco and alcohol users	No use group	Alcohol users	Problem alcohol users	Smokers	Users of all	
		*n* = 114	*n* = 768	*n* = 169	*n* = 329	*n* = 76	*n* = 120	*F (p-*values)^b^
**General family relations**								
Family cohesion	3.03 (.57)	2.85 (.61)_24_	3.08 (.55)_16_	3.00 (.58)	3.09 (.55)_16_	3.0 (.47)	2.76 (.60)_24_	9.16 **
Family conflict	1.73 (.47)	1.88 (.58)_24_	1.68 (.43)_16_	1.68 (.41)_6_	1.70 (.47)_16_	1.79 (.46)	2.02 (.56)_234_	12.81**
Closeness	3.53 (.94)	3.29 (.94)_4_	3.56 (.96)_6_	3.58 (.90)	3.68 (.86)_16_	3.30 (.93)	3.20 (.88)_24_	6.97**
**Change in social relations during Covid-19**								
Fun time with family	2.80 (1.16)	2.61 (1.19)	2.86 (1.15)	2.78 (1.19)	2.87 (1.14)	2.64 (1.20)	2.53 (1.23)	2.97*
Conflicts in family	2.67 (1.56)	2.77 (1.50)	2.52 (1.59)	2.79 (1.50)	2.83 (1.51)	2.58 (1.73)	2.91 (1.57)	3.19*
Meeting with friends	2.39 (1.26)	2.85 (1.17)_23_	2.22 (1.24)_146_	2.18 (1.29)_16_	2.54 (1.23)_2_	2.36 (1.31)	2.78 (1.34)_23_	10.21**
Hanging out without parents’ knowledge	1.79 (1.59)	2.66 (1.49)_234_	1.40 (1.51)_146_	1.69 (1.60)_16_	2.05 (1.59)_1264_	1.97 (1.55)	2.62 (1.40)_234_	26.96**
**Restrictions during Covid-19**								
Following rules and restrictions	3.11 (.66)	2.79 (.67)_23_	3.23 (.61)_146_	3.18 (.63)_16_	3.01(.65)_2_	3.07 (.69)	2.82 (.76)_23_	18.14**

^a^Significant differences between clusters 1–6

^b^*p < .05; **p < .001

In terms of adolescents reporting changes in social relations during the pandemic, the following differences between clusters appeared. Adolescents in the Tobacco and Alcohol Users (1) and Users of All (6) clusters reported more increases in meeting with friends offline than adolescents in the No Use (2) and Alcohol Users (3) clusters. In addition, adolescents in the Problem Alcohol User (4) cluster reported more increases in meeting with friends offline than adolescents in the No Use (2) cluster. Moreover, adolescents in the Tobacco and Alcohol Users (1) and Users of All (6) clusters reported significantly more increases in hanging out without parents’ knowledge than adolescents in the No Use (2), Alcohol Users (3) and Problem Alcohol User (4) clusters. Adolescents in the Problem Alcohol User (4) cluster, however, reported more increases in hanging out without parents’ knowledge than adolescents in the No Use (2) cluster. We found no differences in terms of reported changes in family interactions.

Finally, in terms of attitudes toward restrictions during the Covid-19 pandemic, significant differences appeared among adolescents in the Tobacco and Alcohol Users (1), No Use (2), Alcohol Users (3), Problem Alcohol User (4) and Users of All (6) clusters. Adolescents in the Tobacco and Alcohol Users (1) cluster and Users of All (6) cluster reported less compliance with rules and restrictions in comparison with adolescents in the No Use (2) cluster and Alcohol Users (3) cluster, while adolescents in the Problem Alcohol User (4) cluster reported significantly less compliance with rules during the pandemic than adolescents in No Use (2) cluster.

In sum, across all analyses, adolescents in the Tobacco and Alcohol Users (1) cluster and Users of All (6) cluster reported the lowest levels of general family cohesion and parent–child closeness and highest levels of family conflict. Although adolescents in the Problem Alcohol User (4) cluster also reported higher levels of meeting with friends and lower levels of respecting the rules and restrictions during Covid-19, adolescents in the Tobacco and Alcohol Users (1) cluster and Users of All (6) cluster showed the highest levels of meeting with friends and hanging out without parents’ knowledge during the Covid-19 pandemic, at the same time as they reported the lowest levels of following the rules and restrictions during the Covid-19 pandemic.

## Discussion

This study shows the timely evidence that social relations are playing a role for adolescent substance use during the Covid-19 pandemic. First, we found that adolescents’ reported increase in substance use during the pandemic was predicted by higher levels of general family conflict, increased involvement with peers, and hanging out without parents’ knowledge, as well as a decrease in quality time spent with family and lower levels of compliance with rules and restrictions during this period. Moreover, we discovered six clusters of adolescents with a grouping of scores for adolescent smoking, alcohol use, inebriation, and use of narcotics. The largest cluster of adolescents, i.e., the No Use cluster, either did not use or had decreased use of substances during the Covid-19 pandemic which indicates that most of the adolescents in our sample are handling the situation during the pandemic without substance use initiation or increase. The adolescents in five other clusters, i.e., the Tobacco and Alcohol Users (1), Alcohol Users (3), Problem Alcohol Users (4), Smokers (5), and Users of All (6) clusters, had retained or increased their level of substance use during this period. Further investigations suggested that particularly adolescents in the Tobacco and Alcohol Users (1), Problem Alcohol User (4), and Users of All (6) clusters, thus risk clusters with adolescents who had multiple use of substances and/or inebriation, reported poor parent-adolescent relations, increased peer involvement during the Covid-19 pandemic, and difficulty in conforming to the rules and restrictions during this period.

Healthy family interactions and positive emotional bonds between parents and their adolescents are consistently seen as protective factors of adolescent substance use development [13–17]. During challenging times, when support from other social contexts is limited, family bonds and parental support may be even more critical in terms of adolescents being able to cope with the situation they are in [6]. In addition, having knowledge of what adolescents do and where they go when away from home may prevent adolescents from engaging in misbehavior, including substance use [15]. Not all families, however, are well prepared for challenges induced by the Covid-19 pandemic, which poses a threat for both parents’ and children’s health [26, 27]. It is likely that adolescents who experience a problematic home-situation, including family conflicts and poor family bonds, lack support from family, which makes adolescents inclined to cope with the situation on their own or seek support elsewhere. In addition, if parents do not have knowledge of their adolescents’ whereabouts, adolescents may be at risk of getting involved in problematic behaviors, substance use included [15]. As adolescents and their parents are part of dynamic systems [12], it is also likely that adolescent behavior could have an adverse impact on family interactions and parental support, which yet again poses a risk to adolescent development and an increase in their substance use. Indeed, our findings suggest that conflicted family interactions, as well as a decrease in quality time spent with family added to an increase in time hanging out without parents’ knowledge, could be important risk factors in terms of adolescent substance use during a time of crisis.

Substance use can be a maladaptive strategy to cope with difficult situations [28], which is also evident during the Covid-19 pandemic [4]. In adolescence, substance use often goes hand in hand with peer interactions, parties, and places where no grownups are present [10]. This seems to be the case even during the Covid-19 pandemic, as increased engagement with peers, despite recommendations of social distancing, seems to be one of the risk factors for increases in substance use during the pandemic. It is not surprising that difficulty in following rules and recommendations that include social distancing is yet another risk factor in terms of an increase in adolescent substance use during the pandemic. Indeed, a closer look at the characteristics of adolescents in different substance use clusters revealed that adolescents in the risk clusters reported the highest levels of meeting with friends and hanging out without their parents’ knowledge. In addition, these adolescents had difficulty in conforming to governmental rules to contain the virus. In other words, adolescents who had retained or increased their level of substance use were likely to hang out with peers despite the recommendations of social distancing as a means of containing the virus. Although maintaining social distance may be especially difficult for adolescents, since interaction with friends is an important part of their everyday lives [29], these results are worrisome because peer involvement that includes the use of substances may have an adverse effect on adolescent psychosocial functioning [30] or increase the risks of contracting and spreading the virus [3]. Indeed, substance use, including smoking, seems to be associated with being affected by the Covid-19 virus [31]. In sum, adolescents with retained or increased substance use during the Covid-19 pandemic report a history of strained family interactions and bonds, meeting up with friends during the pandemic and difficulty in conforming to the government’s rules and restrictions to contain the virus, which is a potential threat both to the adolescents themselves and others in their surroundings.

There are some limitations that need attention. The survey was carried out in the early summer of 2020, a season when it is more common for adolescents to meet outside on account of warmer weather, which might have influenced the results. In addition, given the cross-sectional design in the study, we could not control for the level of substance use or family relations prior to the Covid-19 pandemic. Although we reached out to adolescents all over Sweden, there is a question of authenticity of the participants and generalizability of the results as data were collected through social media. This sort of data collection procedure was deemed necessary as traditional survey method (i.e., collecting data in schools) was not possible [32] due to school closure. Finally, given the cross-sectional nature of the data, making causal inferences is not possible. Longitudinal data, preferably with assessment starting before the pandemic and forward, would more accurately offer inferences on directions of effects.

### Practical implications

There are some practical implications of the results. Adolescence is a challenging developmental period, not least during a crisis such as the Covid-19 pandemic. As our findings indicate, adolescents with poor family relations as well as those who have difficulties conforming to rules of social distancing during the outbreak, seem to be at particular risk for substance use during crisis. Providing measures to build adolescent resilience is key. However, as adolescents are a part of multiple systems [12] understanding adolescents and their behavior and providing actions to promote their resilience is barely possible without paying attention to other significant aspects of their developmental systems, such as family, school, and peers. Rather, conforming to recommendations by Masten and Motti-Stefanidi [6], we suggest that it is critical that efforts be directed to the individual *together* with the other systems surrounding them. In that sense, specific efforts could be aimed at adolescents, while also providing support to families and schools that, in turn, would be able to provide social, emotional or psychological support to their adolescents. With the help of this multi-system approach to resilience, public health and social services, as well as other professionals working with adolescents, could identify strategies or interventions to meet the needs of adolescents and their families and provide adequate support to optimize resilience. This would particularly benefit vulnerable adolescents with maladaptive behaviors, such as substance use, who lack support from home, as identified in the current study.

## Conclusions

Even though most of adolescents in our sample seem to manage the situation during the Covid-19 pandemic without the use of tobacco, alcohol or drugs, adolescents could be a vulnerable group that would need close monitoring and support from adults, particularly in the context of school closure and cancellation of other leisure activities. We found that adolescents with generally poor parent–child relationships who report increased involvement with peers and difficulty to conform to rules and regulations during the pandemic are vulnerable to increases of substance use during challenging times. These adolescents need more attention from professionals.

## Data Availability

The datasets used and/or analyzed during the present study are available from the corresponding author on reasonable request.
